# 

**Published:** 1870-01

**Authors:** 


### CONTENTS.

ORIGINAL COMMUNICATIONS.

A Brief Notice of Rev. B. P. Aydelott, M. D., D. D., one of the Founders, and the first President of the Ohio College of Dental Surgery.......... 9

Gold Foil.......... 12

PROCEEDINGS OF SOCIETIES.

Fourth Annual Meeting of the Ohio State Dental Society.......... 15

SELECTIONS.

Removal of Silver Stains from the Hands.......... 40
Carbolic Acid.......... 41
To Clear a room of Mosquitoes.......... 41
A Diamond Test.......... 42
Neuralgia—Sulphate of Nickel.......... 42
To Prevent Death by Chloroform.......... 42
A New Kind of Meerschaum.......... 43
Praiseworthy Action.......... 43

EDITORIAL.

The Mallet in Filling Teeth.......... 44
An Important Invention.......... 45
Kelsey's Improved Vulcanizer.......... 46
Meetings.......... 47
Bills.......... 47
The Rubber Nuisance.......... 48
Professional.......... 49
Acknowledgment.......... 50
Corrections.......... 50

